# A single blood luteinizing hormone level of triptorelin stimulation test can diagnose hypothalamic-pituitary-gonadal axis activation in girls with high body mass index

**DOI:** 10.3389/fped.2025.1629423

**Published:** 2025-08-21

**Authors:** Beilei Zeng, Yinyin Huang, Yuan Zhou, Ye Li, Panwang Huang, Zhuangjian Xu, Yaping Ma

**Affiliations:** ^1^ Department of Pediatrics, Affiliated Hospital of Jiangnan University, Wuxi, Jiangsu, China; ^2^ Wuxi School of Medicine, Jiangnan University, Wuxi, Jiangsu, China

**Keywords:** luteinizing hormone, triptorelin stimulation test, girls, hypothalamic-pituitary-gonadal axis, high body mass index

## Abstract

**Background:**

Body mass index (BMI) may influence peak luteinizing hormone (PLH) levels during gonadotropin releasing hormone (GnRH) or GnRH analogues stimulation testing. BMI effects should be considered when interpreting test results for pubertal disorders in girls with overweight/obesity, but few studies have excluded it.

**Methods:**

This was a hospital data-based retrospective study. Girls with puberty disorders who had been followed up for six months to two years were enrolled in the study. They were divided into the overweight/obesity group and the normal BMI group and all underwent triptorelin stimulation test. Blood samples were collected at 0 min before and 20, 40, and 60 min after the test. Luteinizing hormone (LH) and follicle-stimulating hormone (FSH) serum concentrations were quantified by immunochemiluminometric assay.

**Results:**

A total of 422 girls who underwent 454 triptorelin stimulation tests were included in this study. Among 148 tests performed on 142 overweight/obesity girls, 110 tests were hypothalamic-pituitary-gonadal axis (HPGA) activated and 38 tests were HPGA non-activated. Among 306 tests performed on 284 girls with normal BMI, 214 tests were HPGA activated and 92 tests were non-activated. LH, FSH, and estradiol levels in girls whose HPGA activated were significantly higher than those non-activated. The area under the curves of LH20 min, LH40 min, LH60 min and PLH after triptorelin stimulation tests in girls with overweight/obesity for diagnosing HPGA activation were 0.996, 0.980, 0.990 and 0.994, respectively. There was no statistical significance in the area under the curves between LH20 min, LH40 min, LH60 min and PLH. When LH20 min, LH40 min, LH60 min and PLH were ≥3.26 IU/L, ≥4.09 IU/L, ≥4.27 IU/L and ≥4.51 IU/L, the sensitivity for diagnosing HPGA activation in girls with overweight/obesity were 99.03%, 95.45%, 98.18% and 97.27%, and the corresponding specificity were 94.59%, 97.37%, 100.00%, and 100.00%, respectively. The cut-off value of serum LH60 min after the triptorelin stimulation test for diagnosing HPGA activation in precocious pubertal girls with overweight/obesity was 4.45 IU/L, and in pubertal girls with overweight/obesity was 4.20 IU/L.

**Conclusions:**

LH measurements obtained at 20, 40, or 60 min post-triptorelin stimulation can diagnose HPGA activation in girls with high BMI.

## 1 Introduction

Assessment of the functional status of the hypothalamic-pituitary-gonadal axis (HPGA) is important for the differential diagnosis of diseases related to puberty disorders in girls. The gonadotropin-releasing hormone (GnRH) stimulation test is the laboratory gold standard for determining the functional status of the HPGA (1).

Due to the limited source of GnRH, GnRH analogue (GnRHa) is commonly used instead in clinical practice (2). It has been shown that the triptorelin (one type of GnRHa) stimulation test has high accuracy in the differential diagnosis of central precocious puberty (CPP) and premature thelarche (PT) in girls which provides an effective alternative to the classical GnRH test (3). Moreover, subcutaneous triptorelin is more likely to be well tolerated by children than intravenous GnRH. As body mass index (BMI) increases, the age of breast and pubic hair development is gradually advanced in girls (4). The BMI may influence the peak of gonadotropin (Gn) during GnRH stimulation test in girls. The correlation between BMI and peak luteinizing hormone (PLH) after the GnRH stimulation test is inconclusive, with either a negative or no correlation (5, 6). There are few studies on the value of the triptorelin stimulation test for evaluating the activation of HPGA in abnormal pubertal girls with overweight and obesity. Previous studies have shown that the diagnostic threshold for PLH following GnRHa stimulation test (Diphereline) is lower in overweight and obese girls than in their normal-weight peers. Using a PLH cutoff of 4 IU/L (compared to the traditional 5 IU/L) for diagnosing CPP in this population can improve both sensitivity and specificity (7).

The purpose of the study was to investigate the diagnostic value of a single blood luteinizing hormone (LH) level after triptorelin stimulation test for HPGA activation in pubertal disorders of girls with overweight and obesity.

## 2 Materials and methods

### 2.1 Subjects

We conducted a hospital data-based retrospective study. Among overweight/obesity girls with puberty disorders attending our pediatric endocrine clinic, those who had been followed up for six months to two years were enrolled in the study. Girls with normal BMI during the same period were selected as the control group. This study received an informed consent waiver from our hospital's Ethics Committee in accordance with applicable regulations for retrospective studies.

Criteria for inclusion: girls, proportional physique, normal intelligence, no special clinical signs (e.g., low ear position, skin disease, deafness, visual abnormality, etc.). Triptorelin stimulation test (0.1 mg, subcutaneous injection) was completed at our hospital. No treatment with medication such as GnRH, GnRHa or estrogen that affected the function of HPGA was performed before the stimulation test.

Criteria for exclusion: emaciation, aged <three years, abnormal gene or chromosome testing, clinical syndrome, uterus or ovarian deficiency, hyperthyroidism, Hashimoto's thyroiditis, unknown medical history, etc.

### 2.2 Clinical assessment and experimental methods

Height, weight, BMI, and Tanner stage were recorded on the day of the stimulation test. A calibrated height meter (accurate to 0.1 cm) and an electronic weight scale (accurate to 0.1 kg) were used to measure the height and weight, respectively. Tanner stage was determined by the same pediatric endocrinologist by evaluating the developmental signs of breast, pubic, and axillary hair in girls (8). The more mature side should prevail when the breast stage of the two sides is inconsistent. Orthopantomograms of the left wrist by digital radiography were taken and bone age was calculated by the same pediatric endocrinologist by the TW2R method.

The procedure for the triptorelin stimulation test is as follows. Subjects were injected subcutaneously with triptorelin at 08:30 a.m. (0 min). The two milliliters of blood samples were collected from each of the dorsal veins of the hand at 0, 20, 40, and 60 min of the injection to measure LH and FSH (9). The LH and FSH at 0 min were taken as serum spontaneous LH and spontaneous FSH, respectively. The maximum value of LH and FSH between 0, 20, 40, and 60 min were taken as PLH and peak FSH (PFSH), respectively.

Main reagents and instruments: The triptorelin aqueous injection (Decapeptyl, 0.1 mg, Ferring Pharmaceuticals, Germany) was used for the triptorelin stimulation test. Serum luteinizing hormone (LH), follicle-stimulating hormone (FSH), and estradiol (E2) were measured by ACCESS automated microparticle immunochemiluminescence analyser (DxI800) and accompanying reagents (BECKMAN, USA).

### 2.3 Diagnostic criteria

Diagnostic criteria of CPP: Girls show secondary sexual characteristics before eight years old (1) and follow normal developmental progression. Evidence of the HPGA initiation exists: signs of gonadal development, unilateral ovarian volume >one milliliter on ultrasound, multiple follicles >four millimeters in diameter (10). During follow-up, sexual development continued and linear growth accelerated. Bone age exceeds chronological age by more than one year.

Diagnostic criteria for early puberty: Girls develop secondary sexual characteristics after the age of eight years and before the normal age range for the onset of puberty and have evidence of HPGA initiation.

Diagnostic criteria of peripheral precocious puberty (PPP): Girls have secondary sexual characteristics before age eight years old and there is no evidence of the above suggesting HPGA activation. Girls did not have progress to puberty during follow-up.

Diagnostic criteria of simple breast development: Girls have breast development without accelerated growth, advanced bone age and vaginal hemorrhage. And there is no evidence of HPGA initiation.

Diagnostic criteria for premature pubarche: Appearance of simple pubic hair without development of other secondary sexual characteristics before age eight years in girls (11).

Diagnostic criteria for normal BMI, overweight, and obesity: Referring to the BMI percentile value table for girls aged zero to eighteen years (12), normal BMI is defined as the 5th percentile ≤ BMI < 85th percentile for the same age, overweight is defined as the 85th ≤ BMI < 95th percentile for the same age, obesity is defined as BMI ≥ 95th percentile for the same age.

### 2.4 Statistical analysis

Statistics were performed by SPSS version 26.0. All data were tested for normality by the Kolmogorov–Smirnov method and expressed as median (P25–P75). Comparisons between the two groups of data were performed using the independent samples t-test (when data were normally distributed) and the Mann–Whitney *U*-test (when data were not normally distributed). One-way analysis of variance (ANOVA) and rank Kruskal–Wallis ANOVA (when data were not normally distributed) were used for comparison of multiple data sets. Spearman's correlation was used to describe the correlations between the values of Gn. Differences were considered statistically significant when *P* < 0.05. The area under the curve (AUC) was calculated by the receiver operating characteristic (ROC) curve. And cut-off value, sensitivity, and specificity for diagnosing HPGA initiation were determined based on the highest value of the Youden index:

Youdenindex=sensitivity+specificity−1.

Sensitivity (proportion of true positive tests initiated by HPGA) = number of true positive tests/(number of true positive tests + number of false negative tests).

Specificity (proportion of true negative tests initiated by HPGA) = number of true negative tests/(number of true negative tests + number of false positive tests).

LH and FSH were detected with a sensitivity of 0.2 IU/L. The instrument for E2 has been upgraded to improve the sensitivity from 20 pg/ml to 15 pg/ml. When the actual detected values of LH, FSH, and E2 are lower than their corresponding sensitivity, the corresponding sensitivity values are taken (13).

## 3 Results

### 3.1 General information

**Figure 1:**
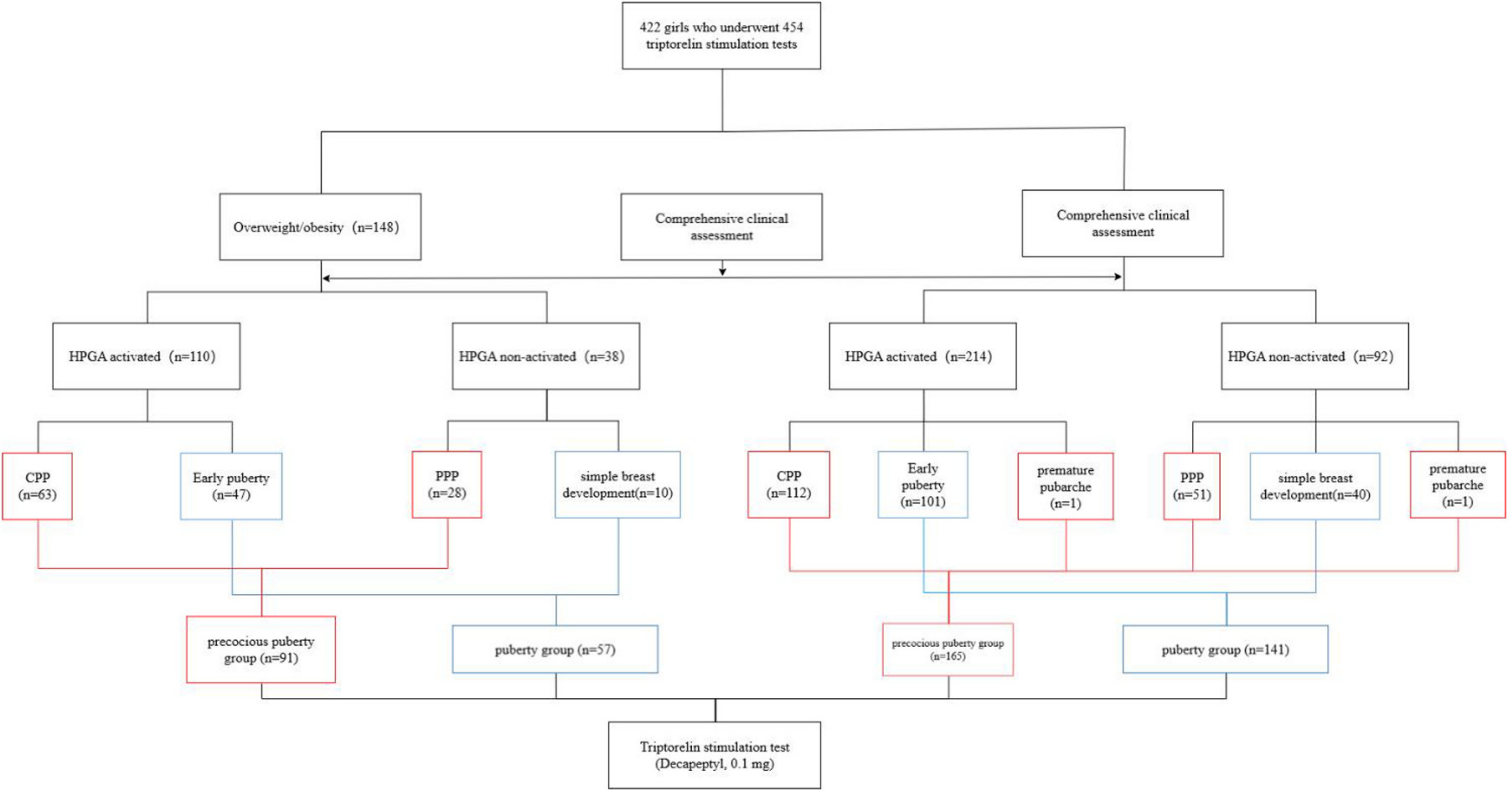
Flowchart onf study participant enrollment.

A total of 422 girls who underwent 454 triptorelin stimulation tests were included in this study (Figure 1). Some subjects underwent repeat tests due to unclear clinical HPGA activation. Among 148 tests performed on 142 girls with overweight/obesity, there were 110 tests of HPGA activated, including 63 tests of CPP and 47 tests of early puberty. There were 38 tests of HPGA non-activated, including 28 tests of PPP and 10 tests of simple breast development. There were 8, 51, 61, 22, and 2 tests in breast Tanner stages Ⅰ, Ⅱ, Ⅲ, Ⅳ, and Ⅴ, respectively, with 4 tests of missing data. The CPP and PPP were classified as the precocious puberty group (*n* = 91), including 63 tests with HPGA activated and 28 tests non-activated. Early puberty and simple breast development were classified as the puberty group (*n* = 57), including 47 tests with HPGA activated and 10 tests non-activated.

Among 306 tests performed on 284 girls with normal BMI, there were 214 tests of HPGA activated, including 112 tests of CPP, 101 tests of early puberty, and 1 test of premature pubarche. There were 92 tests of HPGA non-activated, including 51 tests of PPP, 40 tests of simple breast development, and 1 test of premature pubarche. The test numbers in breast Tanner stages Ⅰ, Ⅱ, Ⅲ, Ⅳ, and Ⅴ were 16, 164, 88, 18, and 7, respectively, with 13 tests of missing data. The CPP, PPP, and premature pubarche were classified as the precocious puberty group (*n* = 165), including 113 tests with HPGA activated and 52 tests non-activated. Early puberty and simple breast development were classified as the puberty group (*n* = 141), including 101 tests with HPGA activated and 40 tests non-activated.

Three girls were overweight at one stimulation test and with a normal BMI at the other test; while there was a girl overweight at two tests, and with a normal BMI at another two tests. Part of clinical characteristics of girls with normal BMI and overweight/obesity are shown in Table 1.

**Table 1:** Clinical characteristics of girls with normal BMI and overweight/obesity.

Group	Age (year)	Height (cm)	Weight (kg)	BMI (kg/m^2^)	Bone age (year)
Normal BMI (*n* = 306)	9.04 (8.22–10.06)	134.90 (129.60–140.83)	28.45 (25.00–32.50)	15.82 (14.91–16.70)	10.72 (9.53–11.31)
Overweight/obesity (*n* = 148)	8.72 (7.90–9.69)	136.55 (132.33–142.18)	36.20 (32.00–41.00)	19.13 (18.15–20.56)	10.82 (10.43–11.40)

Note: Data are expressed as median (P25th, P75th). BMI, body mass index.

### 3.2 Comparison of LH, FSH, and E2 levels between girls with overweight/obesity and normal BMI

**Figure 2:**
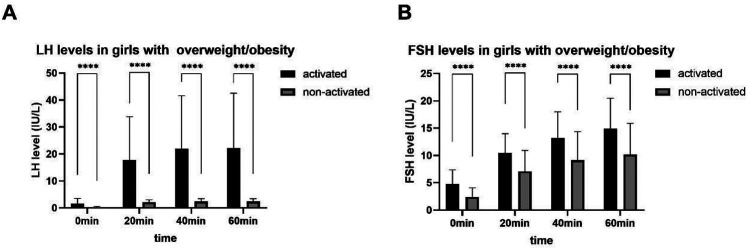
LH **(A)** and FSH **(B)** levels after triptorelin stimulation tests in girls with overweight/obesity. ****, *P* < 0.0001.

In the activated or non-activated HPGA groups, the difference of the LH, FSH, and E2 levels between girls with normal BMI and with overweight/obesity was all not statistically significant (*P* > 0.05). The LH, FSH, and E2 levels in girls with HPGA activated were higher than those with HPGA non-activated, and the difference was statistically significant (*P* < 0.001). The laboratory characteristics are detailed in Table 2 and Figure 2.

**Table 2:** Comparison of LH, FSH, and E2 levels between girls with normal BMI and girls with overweight/obesity.

Index	Non-activated (*n* = 130)				Activated (*n* = 324)			
	Overweight/obesity (*n* = 38)	Normal BMI (*n* = 92)	*Z* value	*P* value	Overweight/obesity (*n* = 110)	Normal BMI (*n* = 214)	*Z* value	*P* value
LH0 min (IU/L)	0.20	0.20	−1.396	0.163	0.89	0.81	−0.413	0.680
	(0.20–0.36)	(0.20–0.27)			(0.28–2.18)	(0.35–2.08)		
FSH0 min (IU/L)	1.97 (1.43–3.05)	2.29 (1.72–3.01)	−1.179	0.238	4.26 (2.96–6.07)	4.22 (2.78–6.04)	−0.765	0.444
LH20 min (IU/L)	2.25 (1.54–2.81)	2.36 (1.72–2.92)	−1.120	0.263	13.09 (5.97–23.71)	13.53 (6.82–26.48)	−0.762	0.446
FSH20 min (IU/L)	6.65	7.13	−1.177	0.239	9.93	9.72	−0.072	0.943
	(4.88–8.40)	(5.47–9.37)			(7.71–11.87)	(7.93–12.23)		
LH40 min (IU/L)	2.40	2.77	−1.390	0.165	16.88	16.20	−0.641	0.521
	(1.66–3.21)	(1.99–3.39)			(7.00–31.26)	(7.85–36.29)		
FSH40 min (IU/L)	9.28	9.09	−1.338	0.181	12.31	13.10	−0.752	0.452
	(6.11–11.25)	(6.96–12.27)			(9.78–15.64)	(9.86–16.83)		
LH60 min (IU/L)	2.39	2.78	−1.538	0.124	16.52	16.88	−0.863	0.388
	(1.73–3.28)	(2.06–3.59)			(7.77–32.61)	(8.33–34.28)		
FSH60 min (IU/L)	10.07	10.99	−1.799	0.072	14.03	14.38	−1.164	0.244
	(6.85–12.23)	(8.00–14.59)			(10.90–17.49)	(11.38–19.01)		
PLH (IU/L)	2.67	2.92	−1.421	0.155	17.63	16.80	−0.766	0.443
	(1.91–3.28)	(2.20–3.59)			(7.98–32.86)	(8.71–37.57)		
PFSH (IU/L)	10.33	11.54	−1.937	0.053	14.23	14.43	−1.061	0.289
	(6.85–12.44)	(8.42–14.62)			(10.96–17.49)	(11.54–19.30)		
PLH/PFSH	0.25	0.24	−0.983	0.326	1.47	1.34	−0.239	0.811
	(0.20–0.38)	(0.18–0.34)			(0.59–2.17)	(0.64–2.14)		
E2 (pg/ml)	20.00	20.00	−0.398	0.691	23.15	26.00	−0.949	0.343
	(15.10–23.50)	(15.00–25.27)			(19.56–40.93)	(20.00–42.87)		

Note: Data are expressed as median (P25th, P75th). LH, luteinizing hormone; FSH, follicle-stimulating hormone; E2, estradiol; HPGA, hypothalamic-pituitary-gonadal axis; PLH, peak LH; PFSH, peak FSH; BMI, body mass index.

### 3.3 ROC analysis of LH for diagnosing HPGA activation in girls with overweight/obesity and normal BMI

#### 3.3.1 AUC of LH level at different time points after triptorelin stimulation tests

**Figure 3:**
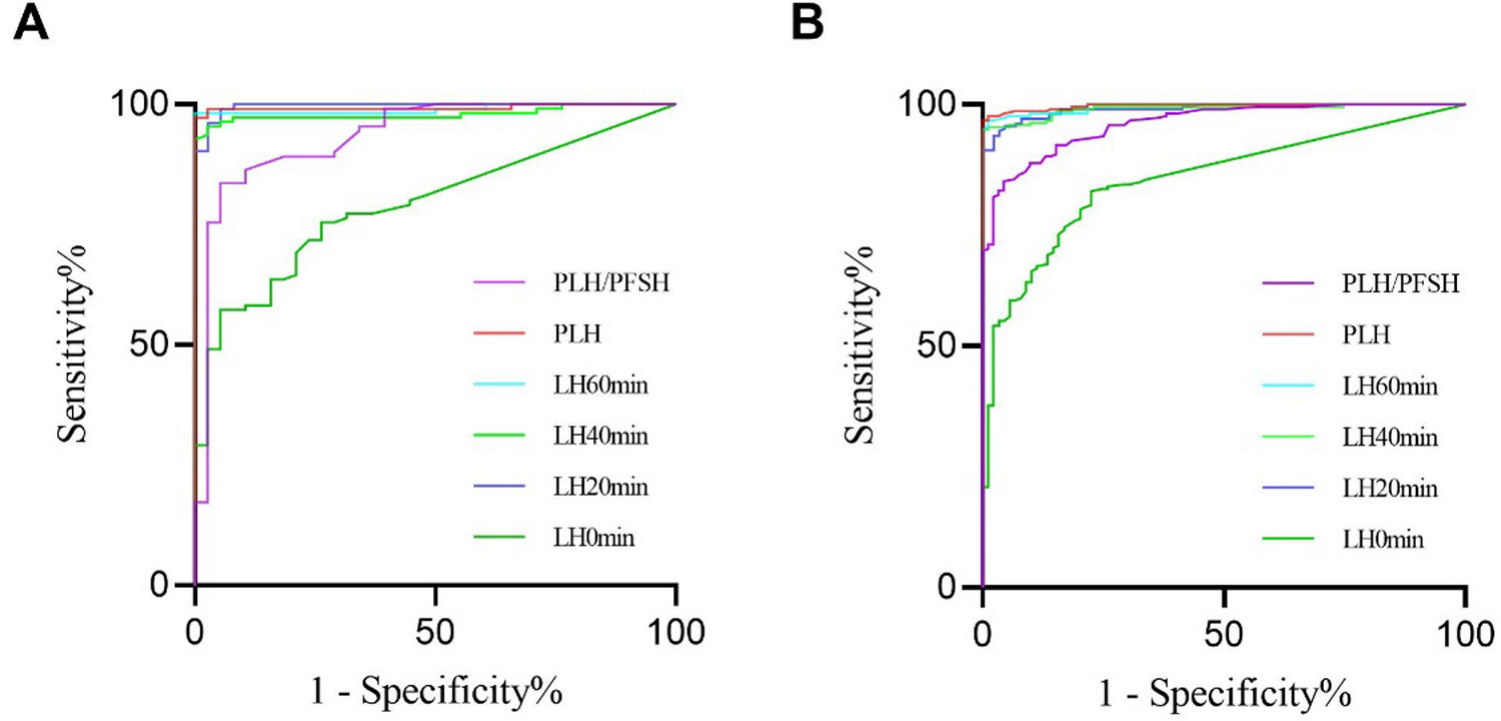
ROC of LH level and PLH/PFSH after triptorelin stimulation tests for diagnosing HPGA activation in girls with overweight/obesity **(A)** and with normal BMI **(B)**.

The AUCs of LH0 min, LH20 min, LH40 min, LH60 min, PLH, and peak luteinizing hormone/peak follicle-stimulating hormone (PLH/PFSH) after triptorelin stimulation tests for diagnosing HPGA activation in girls with overweight/obesity were 0.792, 0.996, 0.980, 0.990, 0.994, and 0.935, respectively (Figure 3A). The AUCs of LH0 min, LH20 min, LH40 min, LH60 min, PLH, and PLH/PFSH after triptorelin stimulation tests for diagnosing HPGA activation in girls with normal BMI were 0.847, 0.990, 0.990, 0.995, 0.997, and 0.961, respectively (Figure 3B). The AUC value of LH0 min, LH20 min, LH40 min, LH60 min, and PLH after triptorelin stimulation tests in diagnosing HPGA activation between the normal BMI and overweight/obese groups were not statistically significant (*P* > 0.05).

#### 3.3.2 Cut-off values, sensitivity, specificity, and AUC comparison of LH at different time points after triptorelin stimulation tests

The cut-off values (sensitivity, specificity) of LH level at 0, 20, 40, and 60 min after triptorelin stimulation tests for diagnosing HPGA activation in girls with normal BMI and overweight/obesity are detailed in Table 3.

**Table 3:** Diagnostic efficacy of LH level and PLH/PFSH for diagnosing HPGA activation in girls.

Group	LH0 min (IU/L)	LH20 min (IU/L)	LH40 min (IU/L)	LH60 min (IU/L)	PLH (IU/L)	PLH/PFSH
Normal BMI (*n* = 306)	0.28 (82.08%, 77.53%)	4.15 (93.50%, 97.70%)	4.70 (94.84%, 100.00%)	4.63 (96.71%, 98.91%)	4.85 (96.73%, 100.00%)	0.49 (84.11%, 95.65%)
Overweight/obesity (*n* = 148)	0.68 (57.27%, 94.74%)	3.26 (99.03%, 94.59%)	4.09 (95.45%, 97.37%)	4.27 (98.18%, 100.00%)	4.51 (97.27%, 100.00%)	0.48 (83.64%, 94.74%)
AUC difference	0.060	−0.004	0.005	0.000	−0.002	0.023
*Z* value	1.344	−0.902	0.472	0.013	−1.139	0.822
*P* value	0.179	0.367	0.637	0.990	0.255	0.411

Note: Data are expressed as cut-off values (sensitivity, specificity). LH, luteinizing hormone; PLH, peak LH; PFSH, peak follicle-stimulating hormone; HPGA, hypothalamic-pituitary-gonadal axis; AUC, area under the curve; BMI, body mass index.

The pairwise comparison of AUC showed that the difference of LH20 min, LH40 min and LH60 min after the triptorelin stimulation test for diagnosing HPGA activation in girls with overweight/obesity was not statistically significant (*P* > 0.05). The pairwise comparison of AUC showed that both LH40 min and LH60 min after the triptorelin stimulation test were able to diagnose HPGA activation in girls with normal BMI, and the difference was not statistically significant (*P* > 0.05), Table 4.

**Table 4:** AUC comparison of LH level at different time points after triptorelin stimulation tests for diagnosing HPGA activation in girls with overweight/obesity and with normal BMI.

Pairs	Overweight/obesity (*n* = 148)			Normal BMI (*n* = 306)		
	AUC difference	*Z* value	*P* value	AUC difference	*Z* value	*P* value
LH0 min—PLH	−0.211	−5.620	<0.001	−0.151	−7.039	<0.001
LH20 min—PLH	−0.003	−1.312	0.189	−0.006	−2.091	0.036
LH40 min—PLH	−0.014	−1.532	0.126	−0.007	−1.671	0.095
LH60 min—PLH	−0.004	−0.921	0.357	−0.002	−1.340	0.180
LH20 min—LH40min	0.011	1.192	0.233	0.001	0.166	0.868
LH20 min—LH60min	0.001	0.145	0.884	−0.004	−1.214	0.225
LH40 min—LH60 min	−0.010	−0.983	0.325	−0.004	−1.125	0.261

Note: AUC, area under the curve; LH, luteinizing hormone; HPGA, hypothalamic-pituitary-gonadal axis; PLH, peak LH; BMI, body mass index.

### 3.4 ROC analysis of LH level for diagnosing HPGA activation in girls with overweight/obesity between the precocious puberty and puberty group

#### 3.4.1 AUC of LH after triptorelin stimulation tests between precocious puberty and puberty group

In the precocious puberty group, the AUCs of LH0 min, LH20 min, LH40 min, LH60 min, PLH, and PLH/PFSH after triptorelin stimulation tests for diagnosing HPGA activation were 0.740, 0.997, 0.998, 0.991, 0.999, and 0.920, respectively. In the puberty group, the AUCs of LH0 min, LH20 min, LH40 min, LH60 min, PLH, and PLH/PFSH after triptorelin stimulation tests for diagnosing HPGA activation were 0.881, 1.000, 0.966, 1.000, 1.000, and 0.968, respectively.

Group comparison results showed that AUC of LH20 min, LH40 min, LH60 min, and PLH after triptorelin stimulation tests in diagnosing HPGA activation for girls between the precocious puberty group and puberty group was not statistically significant (*P* > 0.05).

The cut-off values (sensitivity, specificity) of LH level and PLH/PFSH at 0, 20, 40, and 60 min after triptorelin stimulation tests for diagnosing HPGA activation in girls between the precocious puberty group and puberty group are detailed in Table 5.

**Table 5:** Diagnostic efficacy of LH level and PLH/PFSH for diagnosing HPGA activation in girls with overweight/obesity between the precocious puberty group and puberty group.

Group	LH0 min (IU/L)	LH20 min (IU/L)	LH40 min (IU/L)	LH60 min (IU/L)	PLH (IU/L)	PLH/PFSH
Precocious puberty (*n* = 91)	0.68 (53.57%, 92.59%)	3.81 (100.00%, 96.30%)	4.51 (96.43%, 100.00%)	4.45 (98.21%, 100.00%)	4.75 (98.21%, 100.00%)	0.48 (82.14%, 92.59%)
Puberty (*n* = 57)	0.43 (72.34%, 100.00%)	2.93 (100.00%, 100.00%)	3.85 (95.74%, 100.00%)	4.20 (100.00%, 100.00%)	4.20 (100.00%, 100.00%)	0.42 (87.23%, 100.00%)
AUC difference	−0.141	−0.003	0.032	−0.009	−0.001	−0.048
*Z* value	−2.013	−0.840	1.320	−0.971	−0.588	−1.152
*P* value	0.044	0.401	0.187	0.332	0.556	0.249

Note: Data are expressed as cut-off values (sensitivity, specificity). LH, luteinizing hormone; PLH, peak LH; PFSH, peak follicle-stimulating hormone; HPGA, hypothalamic-pituitary-gonadal axis; AUC, area under the curve.

#### 3.4.2 AUC comparison of LH after triptorelin stimulation tests between precocious puberty and puberty groups

The pairwise comparison of AUC showed that LH20 min, LH40 min, and LH60 min after the triptorelin stimulation test were able to diagnose HPGA activation for girls in the precocious puberty group and in the puberty group, and the difference was not statistically significant (*P* > 0.05), Table 6.

**Table 6:** AUC comparison of LH level at different time points in diagnosing HPGA activation for girls with overweight/obesity between the precocious puberty group and puberty group.

Pairs	Precocious puberty group (*n* = 91)			Puberty group (*n* = 57)		
	AUC difference	*Z* value	*P* value	AUC difference	*Z* value	*P* value
LH0 min—PLH	−0.260	−4.901	<0.001	−0.119	−2.873	0.004
LH20 min—PLH	−0.002	−0.870	0.384	0.000	0.000	1.000
LH40 min—PLH	−0.001	−0.960	0.337	−0.034	−1.391	0.164
LH60 min—PLH	−0.008	−0.977	0.329	0.000	0.000	1.000
LH20 min—LH40min	−0.001	−0.438	0.661	0.034	1.391	0.164
LH20 min—LH60min	0.006	0.697	0.486	0.000	0.000	1.000
LH40 min—LH60 min	0.007	0.925	0.355	−0.034	−1.391	0.164

Note: AUC, area under the curve; LH, luteinizing hormone; HPGA, hypothalamic-pituitary-gonadal axis; PLH, peak LH.

### 3.5 ROC analysis of LH level for diagnosing HPGA activation in girls with normal BMI between the precocious puberty and puberty group

#### 3.5.1 AUC of LH after triptorelin stimulation tests between precocious puberty and puberty group

In the precocious puberty group, the AUCs of LH0 min, LH20 min, LH40 min, LH60 min, PLH, and PLH/PFSH after triptorelin stimulation tests for diagnosing HPGA activation were 0.797, 0.992, 0.994, 0.994, 0.997, and 0.958, respectively.

In the puberty group, the AUCs of LH0 min, LH20 min, LH40 min, LH60 min, PLH, and PLH/PFSH after triptorelin stimulation tests for diagnosing HPGA activation were 0.896, 0.989, 0.986, 0.997, 0.997, and 0.967, respectively. Group comparison showed that AUC of LH20 min, LH40 min, LH60 min, and PLH after triptorelin stimulation tests in diagnosing HPGA activation for girls between the precocious puberty group and puberty group was not statistically significant (*P* > 0.05).

The cut-off values (sensitivity, specificity) of LH level and PLH/PFSH at 0, 20, 40, and 60 min after triptorelin stimulation tests for diagnosing HPGA activation in girls between the precocious puberty group and puberty group are detailed in Table 7.

**Table 7:** Diagnostic efficacy of LH level and PLH/PFSH for diagnosing HPGA activation in girls with normal BMI between the precocious puberty group and puberty group.

Group	LH0 min (IU/L)	LH20 min (IU/L)	LH40 min (IU/L)	LH60 min (IU/L)	PLH (IU/L)	PLH/PFSH
Precocious puberty (*n* = 165)	0.33 (76.11%, 74.00%)	4.16 (93.33%, 98.00%)	4.70 (95.54%, 100.00%)	4.63 (96.43%, 100.00%)	4.66 (98.23%, 100.00%)	0.48 (81.42%, 100.00%)
Puberty (*n* = 141)	0.28 (84.85%, 87.18%)	3.67 (95.79%, 97.30%)	4.54 (95.05%, 100.00%)	4.94 (97.03%, 100.00%)	4.97 (97.03%, 100.00%)	0.55 (87.13%, 95.00%)
AUC difference	−0.099	0.003	0.007	−0.003	0.000	−0.009
*Z* value	−2.231	0.405	0.808	−0.698	0.130	−0.492
*P* value	0.026	0.686	0.419	0.485	0.897	0.623

Note: Data are expressed as cut-off values (sensitivity, specificity). LH, luteinizing hormone; PLH, peak LH; PFSH, peak follicle-stimulating hormone; HPGA, hypothalamic-pituitary-gonadal axis; AUC, area under the curve; BMI, body mass index.

#### 3.5.2 AUC comparison of LH after triptorelin stimulation tests between precocious puberty and puberty groups

The pairwise comparison of AUC showed that LH20 min, LH40 min, and LH60 min after the triptorelin stimulation test were able to diagnose HPGA activation for girls in the precocious puberty group and in the puberty group, and the difference was not statistically significant (*P* > 0.05).

### 3.6 Correlations between LH at different time points and PLH

BMI and PLH were not highly correlated in all cases (*r* = 0.203, *P* < 0.001). Pairwise significant correlations were found between LH0 min, LH20 min, LH40 min, LH60 min, and PLH, respectively. Correlation coefficients between LH at 20, 40, and 60 min after triptorelin stimulation tests and PLH in girls with different BMI were from 0.983 to 0.998, Table 8.

**Table 8:** Correlations between LH0 min, LH20 min, LH40 min, LH60 min, and PLH during triptorelin stimulation tests in girls with different BMI.

	Index	All (*n* = 454)	Normal BMI (*n* = 306)	Overweight (*n* = 87)	Obese (*n* = 61)
LH0 min-PLH	correlation coefficient	0.703	0.718	0.724	0.576
	*P* value	<0.001	<0.001	<0.001	<0.001
LH20 min-PLH	correlation coefficient	0.988	0.988	0.983	0.992
	*P* value	<0.001	<0.001	<0.001	<0.001
LH40 min-PLH	correlation coefficient	0.988	0.987	0.983	0.998
	*P* value	<0.001	<0.001	<0.001	<0.001
LH60 min-PLH	correlation coefficient	0.998	0.998	0.995	0.996
	*P* value	<0.001	<0.001	<0.001	<0.001

Note: LH, luteinizing hormone; PLH, peak LH; BMI, body mass index.

### 3.7 Detection rate of Gn in girls

In this study, LH and FSH detection were conducted in a total of 3,561 tests at 0, 20, 40, and 60 min after the triptorelin stimulation tests, with 133 tests below the lower limit of detection, for a total detection rate of 96.27%. LH detection was conducted in 1,781 tests, with 129 tests below the lower limit of detection, for a detection rate of 92.76%. FSH detection was conducted in 1,780 tests, with 4 tests below the lower limit of detection, for a detection rate of 99.78%.

## 4 Discussion

BMI may affect the onset of HPGA in girls (14). This study included the influence of BMI factors on triptorelin stimulation test and provided a shorter blood collection time point for triptorelin stimulation test, as well as a cut-off value for evaluating precocious puberty and early puberty in girls. The key finding is that LH measurements obtained at 20, 40, or 60 min post-triptorelin stimulation can diagnose HPGA activation in girls with high BMI. Especially, the 60 min post-triptorelin stimulation blood LH level can serve as a laboratory diagnostic biomarker for HPGA activation in pubertal girls with abnormal pubertal development and comorbid overweight or obesity. Specifically, the optimal diagnostic cut-off value of 60 min post-triptorelin stimulation blood LH for identifying HPGA activation is 4.45 IU/L in precocious pubertal girls with overweight/obesity, and 4.20 IU/L in pubertal girls with overweight/obesity. The clinical operation is convenient and worthy of promotion.

The influence of obesity on stimulation tests is controversial. Conventional GnRH/GnRHa stimulation tests may not accurately diagnose HPGA initiation in girls with overweight/obesity. A study for 865 ICPP girls found that BMI had a negative relationship both with PLH and PFSH (5). Previous studies have performed multiple regression analysis of the variables that affect the degree of overweight. A study found that overweight had a positive correlation with Tanner stage for breast development, whereas it had a negative correlation with PLH levels after the gonadorelin stimulation test (6). It indicates that overweight is more likely to exhibit a lower PLH after gonadorelin stimulation compared to normal weight in CPP girls. Rosenfield RL et al. (15) proposed that early pubertal girls with overweight have a marked inactivation of LH. They speculated that excess obesity may subtly inhibit HPGA function in early puberty, as it does in adults, slowing the progression of puberty.

But the complete mechanism about the influence of BMI on Gn secretion in girls with precocious puberty remains unclear. A study has shown that synthetic GnRH distribution volume in obese patients was significantly higher than that in normal subjects (16). The *MKRN3* gene has been reported to be the most common single-gene cause of CPP and plays an important role in the regulation of HPGA (17). *MKRN3* has an inhibitory effect on neuronal GnRH release and prevents activation of the HPGA (18). The lower serum MKRN3 level in CPP girls also confirmed this point, and it was negatively correlated with Gn, E2 and BMI (19, 20). A study has shown that compared with CPP girls of normal weight, obese CPP girls have lower levels of MKRN3, and it is certainly possible that MKRN3 in girls is regulated by nutritional factors or adipokines such as leptin (21). This reveals that patients with obesity and normal BMI have different responses to GnRH, and cut-off values should be set separately to evaluate the onset of HPGA. Body fat percentage might be a factor to consider. Another studies found that the exogenous adipokines (including leptin) contribute to changes in GnRH pulse release by binding to leptin receptors in the hypothalamus (22). Higher levels of leptin, insulin-like growth factor-1 and adiposity in overweight/obese girls with precocious puberty, suggesting a contributory role in pubertal development (23). Excess fat or fat accumulation may affect Gn secretion in girls in puberty, so the LH cut-off value is lower in obese girls. Further researches are needed to explore adipokinetic hormones and their receptors.

Besides, there are different effects of GnRH stimulation tests on precocious puberty and early puberty in obesity girls. Some studies have also pointed out that there is an independent correlation between BMI-SDS and PLH, and the relationship between the two differs in different Tanner stages. A study for 618 ICPP girls found that in early pubertal stage (B2), BMI-SDS was negatively correlated with PLH, yet the correlation between BMI-SDS and PLH changed from negative to positive at the point of 1.5 in middle puberty (B3) and late puberty (B4),which was close to the cut-off value to the definition of obesity (24). A study for 981 ICPP girls found that in Tanner stage 2 and 3 girls, the LH response to GnRH stimulation was clearly influenced by BMI status (6). PLH levels were significantly lower in overweight subjects and lower still in obese subjects. However, PLH levels were not significantly different between the groups in Tanner stage 4 girls. These findings indicated that excess adiposity or fat accumulation might affect Gn secretion in girls at early pubertal stages but not at later pubertal stages. A previous study has shown that the LH concentrations (basal and peak) did not correlate with BMI (25). Our study showed that BMI was not highly correlated with PLH (*r* = 0.203, *P* < 0.001). However, a recent study has pointed out that obese girls have lower PLH than those of normal weight. A study in 634 normal-weight girls and 337 girls with overweight/obesity demonstrated that the cut-off value of PLH for diagnosing CPP in girls with overweight/obesity was lower than that in girls with normal weight with comparable diagnostic accuracy (7), which was consistent with our study. The view was that for obese girls, a lower cut-off of PLH level of 4 IU/L should be considered to diagnose CPP to avoid underdiagnosis. The blood collection time points selected for the test were 60 min, 90 min, and 120 min after the test, which took a long time. Although there were a large number of cases, only cases of precocious puberty were included. Moreover, only the cut-off value of PLH was provided, so multiple blood sampling could not be avoided. Therefore, our study provided a shorter blood collection time point for triptorelin stimulation test, as well as a cut-off value for evaluating precocious puberty and early puberty in girls.

Our results showed that PLH, LH20 min, LH40 min or LH60 min could be used as laboratory diagnostic indicators for HPGA initiation in abnormal pubertal girls (precocious puberty or early puberty) with overweight/obesity, and that PLH, LH40 min or LH60 min could be used as laboratory diagnostic indicators for HPGA initiation in abnormal pubertal girls with normal BMI. In our study, pairwise significant correlations were found between LH0 min, LH20 min, LH40 min, LH60 min, and PLH, respectively. Group comparison showed that AUC of LH0 min, LH20 min, LH40 min, LH60 min, and PLH after triptorelin stimulation tests in diagnosing HPGA activation for girls between the normal BMI group and overweight/obese group was not statistically significant. From the perspective of ROC analysis, triptorelin stimulation test can also diagnose HPGA initiation in girls as well as classical GnRH stimulation test. Our study suggests that triptorelin stimulation test can be used to diagnose HPGA initiation in abnormal pubertal girls with normal BMI, overweight and obesity.

In recent years, there were only a few literatures on blood LH collected at different time points after triptorelin stimulation test to determine the initiation of HPGA in girls with overweight/obesity. Furtherly, our present study explored triptorelin stimulation test within 60 min, which has comparable diagnostic efficacy for HPGA initiation in girls with overweight/obesity and in girls with normal BMI. Freire AV et al. (3) compared the results of the triptorelin stimulation test of 25 girls (5 PT and 20 CPP) with the GnRH stimulation test of 21 girls (8 PT and 13 CPP), including girls with normal weight and with overweight/obesity. The LH level at 180 min after stimulation test could evaluate the HPGA activation state of CPP girls. Cao R et al. (26) studied the triptorelin stimulation test of 1,492 girls with precocious puberty and showed that a single LH60 min could replace the GnRH stimulation test. In that study, 49 cases were complicated with obesity in the ICPP group, compared with 98 cases in the non-CPP group.

The cut-off value for the diagnosis of precocious puberty in overweight/obese girls may be different from that in girls with normal BMI. In several studies of girls with precocious puberty (BMI unknown), the cut-off value of LH after triptorelin stimulation test were 7.65 mUI/L (Immunochemiluminescent analysis) (26), 6 IU/L(automated chemiluminescence assay) (2), and 7 IU/L for immunofluorometric assay or 8 IU/L for electrochemiluminescence immunoassay (3), respectively, to diagnose precocious puberty. A study showed that a cut-off of PLH level of 4 IU/L for obese girls could be considered to diagnose CPP (7), which was lower than 4.2 IU/L of our study. This difference may be due to sample selection, geographical and ethnic differences, different test methods or time points of collecting blood samples.

The cut-off values of LH after triptorelin stimulation test also differ in girls between CPP and early puberty. In girls with overweight/obesity, the cut-off value of LH20 min, LH40 min, LH60 min, and PLH after triptorelin stimulation test for the diagnosis of HPGA initiation in girls with precocious puberty was slightly higher than in those with early puberty. The cut-off value of serum LH60 min after triptorelin stimulation test for diagnosing HPGA activation in precocious pubertal girls with overweight/obesity is 4.45 IU/L, and in pubertal girls with overweight/obesity is 4.20 IU/L. In girls with normal BMI, the cut-off value of LH20 min and LH40 min after triptorelin stimulation test for the diagnosis of HPGA initiation in girls with precocious puberty was slightly higher than in those with early puberty. The cut-off value of LH60 min and PLH for the diagnosis of HPGA initiation in girls with precocious puberty was slightly lower than in those with early puberty. The core of CPP is the premature activation of the HPGA, which results in the pulsing release of GnRH neurons to enhance GnRH. Changes of Gn levels affect the age at which breast development occurs, so the LH cut-off values are different for precocious puberty and early puberty.

In general, this study showed that the cut-off level of LH for diagnosing HPGA activation in girls with overweight/obesity was lower than that in girls with normal BMI. From the AUC comparison, LH60 min has comparable diagnostic efficacy for HPGA initiation in girls with overweight/obesity and normal BMI. The conclusions of this study for obese girls can be generalized.

## 5 Conclusion

A single blood LH level of triptorelin stimulation test can diagnose HPGA activation in girls with high BMI. The cut-off value of blood LH60 min for diagnosing HPGA activation in precocious pubertal girls with overweight/obesity is 4.45 IU/L, and in pubertal girls with overweight/obesity is 4.20 IU/L.

## Data Availability

The original contributions presented in the study are included in the article/Supplementary Material, further inquiries can be directed to the corresponding author/s.

## References

[1] Subspecialty Group of Endocrinologic, Hereditary and Metabolic Diseases, the Society of Pediatrics, Chinese Medical Association; Editorial Board, Chinese Journal of Pediatrics. Consensus statement for the diagnosis and treatment of central precocious puberty (2015). Chin J Pediatr. (2015) 53(6):412–8. 26310550

[2] Poomthavorn P Khlairit P Mahachoklertwattana P. Subcutaneous gonadotropin-releasing hormone agonist (triptorelin) test for diagnosing precocious puberty. Horm Res Paediatr. (2009) 72(2):114–9. 10.1159/000232164 19690429

[3] Freire AV Escobar ME Gryngarten MG Arcari AJ Ballerini MG Bergadá I High diagnostic accuracy of subcutaneous Triptorelin test compared with GnRH test for diagnosing central precocious puberty in girls. Clin Endocrinol. (2013) 78(3):398–404. 10.1111/j.1365-2265.2012.04517.x 22845185

[4] Chen C Zhang Y Sun W Chen Y Jiang Y Song Y Investigating the relationship between precocious puberty and obesity: a cross-sectional study in Shanghai, China. BMJ Open. (2017) 7(4):e014004. 10.1136/bmjopen-2016-014004 28400459 PMC5566589

[5] Fu JF Liang JF Zhou XL Prasad HC Jin JH Dong GP Impact of BMI on gonadorelin-stimulated LH peak in premenarcheal girls with idiopathic central precocious puberty. Obesity. (2015) 23(3):637–43. 10.1002/oby.21010 25645648

[6] Lee HS Yoon JS Hwang JS. Luteinizing hormone secretion during gonadotropin-releasing hormone stimulation tests in obese girls with central precocious puberty. J Clin Res Pediatr Endocrinol. (2016) 8(4):392–8. 10.4274/jcrpe.3091 27215137 PMC5197996

[7] Sakornyutthadej N Mahachoklertwattana P Wankanit S Poomthavorn P. Peak serum luteinising hormone cut-off during gonadotropin-releasing hormone analogue test for diagnosing central precocious puberty was lower in girls with obesity as compared with girls with normal weight. Clin Endocrinol. (2024) 100(4):368–78. 10.1111/cen.15026 38300440

[8] Marshall WA Tanner JM. Variations in pattern of pubertal changes in girls. Arch Dis Child. (1969) 44(235):291–303. 10.1136/adc.44.235.291 5785179 PMC2020314

[9] Ma Y Nie S Ben Z Xu Z Zhou H Zhao J The dynamic trends of urinary LH and FSH assayed by ICMA during triptorelin stimulation tests in girls—a pilot study. Clin Lab. (2018) 64(10):1701–8. 10.7754/Clin.Lab.2018.180514 30336539

[10] Adolescent Health Care Group of Women Health Care Society, China Preventive Medicine Association. Consensus on diagnosis and treatment of female precocious puberty. Chin J Woman Child Health Res. (2018) 29(2):135–8. 10.3969/j.issn.1673-5293.2018.02.001

[11] Cavarzere P Mauro M Vincenzi M Lauriola S Teofoli F Gaudino R Children with premature pubarche: is an alterated neonatal 17-Ohp screening test a predictive factor? Ital J Pediatr. (2018) 44(1):10. 10.1186/s13052-018-0444-6 29338783 PMC5771218

[12] The Subspecialty Group of Endocrinologic, Hereditary and Metabolic Diseases, The Society of Pediatrics, Chinese Medical Association. Guidelines for diagnosis and treatment of children with short stature. Chin J Pediatr. (2008) 46(6):428–30. 19099778

[13] Resende EAMR Lara BHJ Reis JD Ferreira BP Pereira GA Borges MF. Assessment of basal and gonadotropin-releasing hormone-stimulated gonadotropins by immunochemiluminometric and immunofluorometric assays in normal children. J Clin Endocrinol Metab. (2007) 92(4):1424–9. 10.1210/jc.2006-1569 17284632

[14] Wang Y Gou H Guo J. Risk factors for precocious puberty: a systematic review and meta-analysis. Psychoneuroendocrinology. (2025) 176:107427. 10.1016/j.psyneuen.2025.107427 40081314

[15] Rosenfield RL Bordini B. Evidence that obesity and androgens have independent and opposing effects on gonadotropin production from puberty to maturity. Brain Res. (2010) 1364:186–97. 10.1016/j.brainres.2010.08.088 20816944 PMC2992573

[16] Chikamori K Suehiro F Ogawa T Sato K Mori H Oshima I Distribution volume, metabolic clearance and plasma half disappearance time of exogenous luteinizing hormone releasing hormone in normal women and women with obesity and anorexia nervosa. Acta Endocrinol. (1981) 96(1):1–6. 10.1530/acta.0.0960001 7006293

[17] Chen ZQ Zhang XR. MKRN3 Regulates central precocious puberty in children. Chin J Biochem Mol Biol. (2025):41(1):99–104. 10.13865/j.cnki.cjbmb.2024.10.1128

[18] Palumbo S Cirillo G Aiello F Papparella A Miraglia Del Giudice E Grandone A. MKRN3 Role in regulating pubertal onset: the state of art of functional studies. Front Endocrinol. (2022) 13::991322. 10.3389/fendo.2022.991322 PMC9523110 36187104

[19] Grandone A Cirillo G Sasso M Capristo C Tornese G Marzuillo P MKRN3 Levels in girls with central precocious puberty and correlation with sexual hormone levels: a pilot study. Endocrine. (2018) 59(1):203–8. 10.1007/s12020-017-1281-x 28299573

[20] Hagen CP Sørensen K Mieritz MG Johannsen TH Almstrup K Juul A. Circulating MKRN3 levels decline prior to pubertal onset and through puberty: a longitudinal study of healthy girls. J Clin Endocrinol Metab. (2015) 100(5):1920–6. 10.1210/jc.2014-4462 25695892

[21] Eren SE Şimşek E. Comparison of makorin ring finger protein 3 levels between obese and normal weight patients with central precocious puberty. J Clin Res Pediatr Endocrinol. (2023) 15(2):182–9. 10.4274/jcrpe.galenos.2023.2022-6-6 36728292 PMC10234051

[22] Soliman A Yasin M Kassem A. Leptin in pediatrics: a hormone from adipocyte that wheels several functions in children. Indian J Endocr Metab. (2012) 16(9):577. 10.4103/2230-8210.105575 PMC3602987 23565493

[23] Kang MJ Oh YJ Shim YS Baek JW Yang S Hwang IT. The usefulness of circulating levels of leptin, kisspeptin, and neurokinin B in obese girls with precocious puberty. Gynecol Endocrinol. (2018) 34(7):627–30. 10.1080/09513590.2017.1423467 29303010

[24] Zhao Y Hou L Gao HJ Zhan D Zhang C Luo XP. Independent relationship between body mass index and LH peak value of GnRH stimulation test in ICPP girls: a cross-sectional study. J Huazhong Univ Sci Technol [Med Sci]. (2017) 37(4):556–62. 10.1007/s11596-017-1772-2 28786058

[25] Giabicani E Allali S Durand A Sommet J Couto-Silva AC Brauner R. Presentation of 493 consecutive girls with idiopathic central precocious puberty: a single-center study. PLoS One. (2013) 8(7):e70931. 10.1371/journal.pone.0070931 23936254 PMC3728106

[26] Cao R Liu J Fu P Zhou Y Li Z Liu P. The diagnostic utility of the basal luteinizing hormone level and single 60-Minute post GnRH agonist stimulation test for idiopathic central precocious puberty in girls. Front Endocrinol. (2021) 12:713880. 10.3389/fendo.2021.713880 PMC8387794 34456870

